# 520 Efficacy of high volume hemofiltration (HVHF) with step-down approach in critically ill burn patients

**DOI:** 10.1093/jbcr/irac012.151

**Published:** 2022-03-23

**Authors:** Anjay Khandelwal, Richard B Lou, Rupesh Raina

**Affiliations:** Akron Children's Hospital, Akron, Ohio; Akron Children's Hospital, Akron, Ohio; Akron Children's Hospital; Cleveland Clinic; Akron Nephrology Associates, Akron, Ohio

## Abstract

**Introduction:**

Continuous renal replacement therapy (CRRT) is the standard of care for critically ill burn patients with acute kidney injury. Historical mortality rates for burn patients requiring CRRT is over 50%. High volume hemofiltration (HVHF) is a purification method that may have superior outcomes in the critically ill but has not been well studied in burn patients. We report results of utilizing HVHF with a step-down approach in our burn center.

**Methods:**

Retrospective review of all adult burn patients at a verified adult and pediatric burn center requiring continuous veno-venous hemodiafiltration (CVVHDF) from 2018 to 2021 when HVHF (50ml/kg/hr) for 18-24 hours with a step-down approach (25-30ml/kg/hr) was started. A normocarbia bicarbonate based dialysate was used with blood flow rates of 200ml/hr along with citrate anticoagulation. Demographics, indications and duration of CVVHDF, duration of vasopressor requirements, days on mechanical ventilation, length of stay, correction of acidosis and mortality were recorded.

**Results:**

Thirteen patients were identified, of which four were excluded as they had an exfoliative skin disease (two patients) or were treated with conventional CVVHDF (two patients). Of the remaining nine patients (six male and three female) the average age was 58.7 years (range: 42-75) and average total body surface area burn (%TBSA) was 46% (range: 20-92). All patients were diagnosed with AKI (Acute Kidney Injury Network [AKIN] Class III) with the indication for CRRT being diuretic resistant fluid overload in three patients and sepsis in the remaining six patients. Of the three patients with fluid overload, two developed sepsis while on CRRT. Acute respiratory distress syndrome (ARDS) as defined by the Berlin criteria was noted in six patients (66%). Three patients died (33% mortality) with the remaining six being eventually converted to intermittent hemodialysis and progressing to renal recovery. Amongst survivors, the duration of CRRT ranged between 7-33 days (average 13.8 days) and duration of vasopressor support from 1-30 (average 17 days). Metabolic acidosis resolved in all survivors within 48 hours. No hemorrhagic events were noted and all patients tolerated regional citrate anticoagulation well. Reduction in serum creatinine was noted to be significant.

**Conclusions:**

High volume hemofiltration resulted in improved mortality rates compared to what is reported in the literature. Further research and comparative studies are required to determine if HVHF with a step-down approach is superior.

